# The impact of the COVID-19 pandemic on acute appendicitis patients in a tertiary care center in Lebanon

**DOI:** 10.1186/s12893-023-02273-3

**Published:** 2024-01-08

**Authors:** Joelle Hassanieh, Nader Zalaquett, Alicia Khazzeka, Ryan El Ghazal, Mansour Riachi, Salim Habib, Ahmad Zaghal

**Affiliations:** 1 Department of Surgery, American University of Beirut-Medical Center, Beirut, Lebanon; 2 Department of Anatomy, Cell Biology, and Physiological Sciences, Faculty of Medicine and Medical Center, American University of Beirut, Beirut, Lebanon

**Keywords:** COVID-19, Acute Appendicitis, Pandemic, Surgery

## Abstract

**Introduction:**

With it becoming a global pandemic, the coronavirus disease of 2019 (COVID-19) imposed public health restraints that hampered patient’s presentation to hospitals. In Lebanon, little is known about the presentation patterns of acute appendicitis (AA) patients among different population groups during the COVID-19. Therefore, this study aims to assess the effects of the COVID-19 pandemic on the rates of cases seen during the pandemic period, the adopted management strategies, and evaluate the patient outcomes compared to presentations from previous years.

**Methods:**

This is a retrospective, observational cohort study with no interventional procedures. All patients presented to our tertiary health care center were diagnosed with AA between February 2019 and February 2021 comprised the study analysis. We divided our patients into the pre-pandemic period cohort March 1st, 2019, till February 29th, 2020, and the pandemic period cohort March 1st 2020 till March 1st 2021.

**Results:**

We collected data retrospectively from 342 patients: 201 patients presented in the pre-pandemic period and 141 during the pandemic period. Male predominance was seen in both cohorts (51.7% and 58.9% respectively). A decrease in the number of AA cases was seen during the pandemic, however, the duration of symptoms before presentation was similar in both cohorts (*p* = 0.382) Additionally, the number of complicated appendicitis cases was not different between cohorts. The main imaging modality was CT in both groups with no statistically significant difference in the type of imaging between them (*p* = 0.398). Further, the predominant treatment modality remained surgery during the pandemic, with no difference between both periods (*p* = 0.200), and no statistically significant difference in the type of surgery performed as laparoscopic surgery remained the most common surgery type in the pandemic period (*p* = 0.43). Finally, no extra surgical and post-surgical complications were identified.

**Conclusion:**

In conclusion, our study is an example of how the COVID-19 pandemic did not significantly affect patients presenting for AA. Despite the COVID-19-related restrictions, Lebanese patients with worrying symptoms were presenting to the emergency department and the American University of Beirut Medical Center was providing them with the standards of care. Our study mirrors the Lebanese experience and gives an example of a population that focused more on their current symptoms than the fear of acquiring the COVID-19 virus. Further research is needed to assess whether this was the correct approach during these times.

## Introduction

With it becoming a global pandemic, the corona virus disease of 2019 (COVID-19) imposed public health restraints that hampered patients’ presentations to hospitals. Patients have deferred hospital admissions and emergency department (ED) visits due to the rising concerns of contracting the COVID-19 virus [1]. Acute appendicitis (AA) patients were affected as such.

With the virus emergence and the pandemic onset, findings from scientific literature have revealed that patients presenting to hospitals with progressed stages of appendicitis may be due to delayed medical attention [2]. Acute appendicitis is one of the most common causes of acute abdomen and is the most frequent surgical disease for patients presenting to emergency departments worldwide [3]. It is a medical condition unlikely to improve without medical or surgical intervention [4].

A recent study authored by Romero J, et al. have reported a significant severity of diagnosed appendicitis cases throughout the pandemic period in Columbia [5]. Another study conducted by Rosenthal, Martin G. et al. reported that acute appendicitis presentations to a large hospital network have sharply decreased during the COVID-19 pandemic, whereas the management approach and patient outcomes have not changed from previous years [6]. In their research paper, Velayos M et al. reported that there existed a higher post-operative complication rate, and longer hospital stays with indifference in regards to the onset of symptoms during COVID-19 [7].

It is obvious that COVID-19 uniquely affects the healthcare system worldwide. In Lebanon, little is known about the presentation patterns of acute appendicitis patients among different population groups during the COVID-19 pandemic considering that Lebanon is currently suffering from an economic collapse in parallel. Therefore, the purpose of this study is to assess the effects of the COVID-19 pandemic on the rates of AA cases seen during this period, the adopted management strategies, and at a final stage to evaluate the patient outcomes as compared to presentations from previous years.

### Study hypothesis

We hypothesize that there will be a lower patient presentation yet worse disease severity and greater post-management complications during the pandemic period due to the mandated lockdown and fear of contracting the covid in high-exposure settings as our tertiary health care center. We also hypothesize that COVID-19 has restricted people’s gathering thus decreasing the infectious transmission rate among patients, particularly kids, hence diminishing the incidences of acute appendicitis.

## Materials and methods

This is a retrospective, observational cohort study with no interventional procedures. Post Institutional Review Board (IRB) approval, patient files and medical charts were screened. All patients, regardless of age, who presented to our tertiary health care center diagnosed with acute appendicitis between February 2019 and February 2021 comprised the study analysis. Inclusion criteria entailed patients presenting to any hospital unit for abdominal pain, acute appendicitis, during the study period. Patients with missing data and those lost to follow up were excluded.

Data were compared for the same period between the year of the pandemic to the earlier year with no pandemic. We divided our patients into the pre-pandemic period cohort March 1st, 2019, till February 29th, 2020, and the pandemic period cohort March 1st, 2020, till March 1st, 2021. Patient charts were accessed via electronic medical record. Data collection was restricted primarily to those variables necessary to define clinical patient characteristics.

This study can provide a better understanding of the impact of the pandemic on the clinical presentation, outcomes, and complications of patients with a diagnosis of acute appendicitis. There were no risks associated with this study. No direct benefits were imparted to the enrolled participants. No direct monetary compensation was provided to the enrolled participants.

### Statistical analysis

Patient baseline data were compared between the pandemic and the pre-pandemic period. The Fisher’s exact probability test or the Chi-square test was applied for categorical data, or the students t-test for continuous variables. Categorical data were presented as N (%) as compared to continuous data that were reported as Mean and Standard Deviation.

## Results

A total of 374 patients with an initial diagnosis of appendicitis were admitted to the American University of Beirut Medical Center (AUBMC) between March 1st of 2019 and March 1st of 2021. A total of 342 cases remained eligible for analysis. On admission, almost all the patients were COVID negative (339 patients (99.1%)).

The patients’ baseline data is listed in Table 1. The mean age of participants was reported to be 31.78 ± 18.76 years, with a non-statistically significant lower mean age in patients presenting in the pandemic period (32.59 vs. 32.61, *p* = 0.336). Male predominance was observed in both cohorts of the studied sample with 51.7% and 58.9% males in the pre-pandemic and pandemic periods respectively (*p* = 0.193). Patient comorbidities included but were not limited to hypertension 16(4.7%), dyslipidemia 14(4.1%), malignancy 10(2.9%), and diabetes 7(2%) were the most reported comorbidities, with no statistical significance between our studied groups (*p* = 0.087).

**Table 1:** Participant Baseline Information

Patient Baseline Data (N = 342)		Pre-Pandemic Period (N = 201)	Pandemic Period (N = 141)	*p*-value
Characteristics	Mean ± Standard Deviation (S.D)			
Age	31.78 ± 18.76	32.61 ± 18.65	30.59 ± 18.94	0.336
Gender				
Males	187(54.7%)	104(51.7%)	83(58.9%)	0.193
Females	155(45.3%)	97(48.3%)	58(41.1%)	
Medical History				
Comorbidities				
Diabetes	7(2%)	3(1.5%)	4(2.8%)	0.087
Hypertension	16(4.7%)	7(3.5%)	9(6.4%)	
Malignancy	10(2.9%)	8(4%)	2(1.4%)	
Cardiovascular Disease	7(2%)	6(3%)	1(0.7%)	
COPD and Cardiovascular Disease	3(0.9%)	2(1%)	1(0.7%)	
Dyslipidemia	14(4.1%)	11(5.5%)	3(2.1%)	
Obesity	3(0.9%)	3(1.5%)	0(0%)	
Appendicitis	7(2%)	6(3%)	1(0.7%)	
Combined (Diabetes, Hypertension, Dyslipidemia)	12(3.5%)	6(3%)	6(4.3%)	
Familiar Mediterranean Fever (FMF)	2(0.6%)	1(0.5%)	1(0.7%)	
Other	82(24%)	54(26.9%)	27(19.1%)	
None	179(52.3%)	92(45.8%)	86(61%)	

Patient symptoms at presentation were detailed as follows (Table 2): Three-hundred thirty (96.5%) patients presented with abdominal pain, 168 (49.1%) with nausea, 129 (37.7%) with vomiting, 91 (26.6%) patients with fever, 46 (13.5%) patients with decreased appetite, 41(12%) with diarrhea, 21 (6.1%) patients with constipation. A single patient (0.3%) diagnosed during the pandemic period was reported to be symptom free. Mean duration of symptoms was 44.21 hours, approximately equivalent to two days.

**Table 2:** History, physical exam, and blood testing

Diagnosis (N = 342)		Pre-Pandemic Period (N = 201)	Pandemic Period (N = 141)	*p*-value
**Symptoms at Presentation**				
Abdominal Pain	330(96.5%)	193(96%)	137(97.2%)	0.767
Nausea	168(49.1%)	98(48.8%)	70(49.6%)	0.871
Vomiting	129(37.7%)	77(38.3%)	52(36.9%)	0.788
Diarrhea	41(12%)	25(12.4%)	16(11.3%)	0.760
Constipation	21(6.1%)	13(6.5%)	8(5.7%)	0.763
Decreased Appetite	46(13.5%)	22(10.9%)	24(17.0%)	0.105
Fever	91(26.6%)	59(29.4%)	32(22.7%)	0.170
None	1(0.3%)	0(0%)	1(0.7%)	0.412
Mean Duration of Symptoms (Hours)	44.21	46.83	40.58	0.382
**Physical Exam Findings**				
Abdominal Tenderness	288(84.2%)	171(85.4%)	117(83.0%)	0.601
Rovsing’s Sign	58(17%)	34(16.9%)	24(17%)	0.980
Rebound Tenderness	101(29.5%)	62(30.8%)	39(27.7%)	0.525
Psoas Sign	25(7.3%)	15(7.5%)	10(7.1%)	0.897
Guarding	47(13.7%)	28(13.9%)	19(13.5%)	0.904
McBurney Point Tenderness	211(61.7%)	121(60.2%)	90(63.8%)	0.497
Murphy Sign	10(2.9%)	8(4%)	2(1.4%)	0.206
Markle Sign	1(0.3%)	0	1(0.7%)	0.412
Obturator Sign	22(6.4%)	14(7%)	8(5.7%)	0.632
No Findings	31(9.1%)	16(8%)	14(9.9%)	0.526
**Inflammatory Markers Presentation**				
**White Blood Cell (WBC) Count (%)**				
Neutrophils	76.61 ± 12.82	76.65 ± 11.97	76.42 ± 13.99	0.816
Lymphocytes	15.82 ± 10.29	15.48 ± 9.17	16 ± 11.72	0.483
Monocytes	6.55 ± 2.75	6.49 ± 2.846	6.63 ± 2.632	0.655
Eosinophils	1.56 ± 1.66	1.53 ± 1.600	1.61 ± 1.770	0.702

Physical exam findings were consistent in both groups, with abdominal tenderness (83.6%) being the most frequent symptom, followed by McBurney point tenderness (61.1%) and rebound tenderness (29.5%) respectively. Neutrophils accounted for 76.65%, lymphocytes for 15.48%, monocytes for 6.49%, and eosinophils for 1.53% of the white blood cells in pre-pandemic patients, compared to 76.42%, 16.30%, 6.63%, 1.61% respectively in pandemic patients.

Three imaging modalities were used for the diagnosis of acute appendicitis (Table 3): ultrasound (US), computed tomography (CT), and magnetic resonance imaging (MRI). Most patients were diagnosed using computed tomography CT scans with a total of 299 patients accounting for 87.4% of the patients. Both CT and Ultrasound were performed on 21 (6.1%) patients (*p* = 0.398). The CT findings reported evidence of peri-appendicular abscess in 20 (6.4%) patients, perforation in 28 (8.9%) patients (*p* = 1.00). Evidence of peri-appendicular fluid findings and appendicolith was documented in 103 (32.9%) patients and 79 (25.2%) respectively. These patients exhibited thickening of the appendix wall [218 (69.6%)]. The diameter of the appendix varied with 249 (79.6%) of patients having a diameter larger than 7 mm, while only 11 (3.5%) having a diameter below 7 mm and 24 (7.7%) having exactly 7 mm according to CT findings (*p* = 0.749). As for the patients diagnosed using ultrasound modality, the diameter of the appendix varied with 19 (51.4%) of patients having a diameter larger than 7 mm, while only 4 (10.8%) having a diameter below 7 mm and 5 (13.5%) having exactly 7 mm (*p* = 0.781).

**Table 3:** Diagnostic Imaging

Imaging Modalities (N = 342)		Pre-Pandemic Period (N = 201)	Pandemic Period (N = 141)	*P*-value
CT	299(87.4%)	174(86.6%)	125(88.7%)	0.398
Ultrasound	21(6.1%)	13(6.5%)	8(5.27%)	
CT and Ultrasound	14(4.1%)	11(5.5%)	3(2.1%)	
MRI and Ultrasound	2(0.6%)	1(0.5%)	1(0.7%)	
None	6(1.8%)	2(1%)	4(2.8%)	
**CT Findings (N = 313)**				
Evidence of Peri-appendicular abscess	20(6.4%)	13(7%)	7(5.5%)	0.419
Evidence of Perforation	28(8.9%)	17(9.2%)	11(8.6%)	0.478
Evidence of Peri-appendicular fluid	103(32.9%)	64(34.6%)	39(30.5%)	0.375
Evidence of appendicolith	79(25.2%)	43(23.2%)	36(28.1%)	0.289
Thickening of the appendix wall	218(69.6%)	132(71.4%)	86(67.2%)	0.718
**Diameter of appendix**				
Above 7 mm	249(79.6%)	150(81.1%)	99(77.3%)	0.749
Below 7 mm	11(3.5%)	5(2.7%)	6(4.7%)	
Exactly 7 mm	24(7.7%)	14(7.6%)	10(7.8%)	
Ultrasound Findings (N = 37)				
Evidence of Peri-appendicular abscess	1(2.7%)	1(4%)	0(0%)	1.00
Evidence of Perforation	2(5.4%)	1(4%)	1(8.3%)	1.00
Evidence of Peri-appendicular fluid	13(35.1%)	9(36.0%)	4(33.3%)	1.00
Evidence of appendicolith	4(10.8%)	2(8%)	2(16.7%)	0.582
Thickening of the appendix wall	23(62.2%)	14(56.0%)	9(75%)	0.306
Diameter of appendix				
Above 7 mm	19(51.4%)	13(52%)	6(50%)	0.781
Below 7 mm	4(10.8%)	3(12%)	1(8.3%)	
Exactly 7 mm	5(13.5%)	2(8%)	3(25%)	

Two treatment modalities were conducted with predominance of surgical approach 304(88.9%) compared to medical 38 (11.1%) treatment (*p* = 0.200) (Table 4). The medical treatment consisted of one antibiotic regimen. 29 patients in pre-pandemic period and 9 patients in pandemic period were treated using this approach. The antibiotic regimen did not vary between the different periods and consisted of mostly penicillins, mainly amoxicillin-clavulanate, followed by fluoroquinolones, such as ciprofloxacin, levofloxacin, and moxifloxacin. All antibiotic regimens were administered for 5 to 15 days, depending on the severity of each case. In addition, one patient was treated with ceftizoxime, a third-generation cephalosporin, and two patients were treated with carbapenems, meropenem and ertapenem, in conjunction with tazocin. Lastly, metronidazole was used in three patients as adjunct to ciprofloxacin.

**Table 4:** Treatment Context

Treatment Modality		Pre-Pandemic Period (N = 201)	Pandemic Period (N = 141)	*p*-value
Surgical	304 (88.9%)	175(87.1%)	129(91.5%)	0.200
Medical	38 (11.1%)	26(12.9%)	12(8.5%)	
**Medical Treatment**				
Mean Duration of antibiotic treatment (days)	4.26	4.26	4.25	0.971
**Surgical Treatment**				
Types of Surgery (N = 342)		N = 201	N = 141	
Open	39(11.4%)	21(10.4)	18(12.8%)	0.430
Laparoscopic	263(76.9%)	153(76.1%)	110(78%)	
No Surgery	40(11.7%)	27(13.4%)	13(9.2%)	
Drain Placement				
Yes	33(9.6%)	19(9.5%)	14(9.9%)	0.491
No	269(78.7%)	155(77.1%)	114(80.9%)	
Mean Duration of Surgery	74.09	75.09	72.74	0.512
**Surgical Findings**				
Intra-operative evidence of abscess	22(6.4%)	14(7%)	8(5.7%)	0.411
Intra-operative evidence of perforation	47(13.7%)	27(13.4%)	20(14.2%)	0.490
Intra-operative finding of gangrenous	17(5%)	9(4.5%)	8(5.7%)	0.452

The most frequently performed surgical procedure in both complicated (91%) and non-complicated appendicitis (100%) was the conventional 3-port laparoscopic approach (*p* = 0.430). Drain was placed in 33 (9.6%) patients (*p* = 0.491). The duration of surgery was 74.09 ± 30.732 min. As for intraoperative findings, evidence of abscess and perforation was found in 22 (6.4%) (*p* = 0.411) and 47 (13.7%) of patients respectively (*p* = 0.490). Lastly, intraoperative finding of gangrenous tissue was found in 17(5%) of patients with no reported significance between both studied groups (*p* = 0.452).

Forty patients (11.7%) of the studied cohort were documented to manifest complications during their admission. There was no significant change in the number of complicated admissions between the pre-pandemic and pandemic phase (11.4% vs. 12.1%, *p* = 0.866). Additionally, no significant patient reoperation procedures (1 vs. 1.4%) or post-operative abscesses outcome (2 vs. 2.8%) were seen in the records of both patient cohorts respectively (Table 5). Thirty-day post-discharge admissions were documented in 27 (7.9%) of our studied patient sample (*p* = 0.645). Most of these patients 321(93.9%) did not require re-admission related to acute appendicitis management (*p* = 0.763) (Table 6). Reasons for re-admission included wound infection, fluid collection on CT, recurrent fever and abscess formation requiring IR drainage, appendicitis recurrence, ruptured appendicitis, diabetic ketoacidosis, hematuria, and right sided axillary adenopathy.

**Table 5:** Hospital Stay

Patient Hospital Stay Status		Pre-Pandemic Period	Pandemic Period	*p*-value
**Complications During Admission**				
Yes	40(11.7%)	23(11.4%)	17(12.1%)	0.866
**Patient Re-operation**				
Yes	4(1.2%)	2(1%)	2(1.4%)	1.000
**Post-op Abscess**				
Yes	8(2.3%)	4(2%)	4(2.8%)	0.337

**Table 6:** Patient Follow-up

Post- Discharge 30-day admission		Pre-Pandemic Period	Pandemic Period	*p*-value
Yes	27(7.9%)	17(8.5%)	10(7.1%)	0.645
**Re-admission Related to Acute Appendicitis Management**				
Yes	21(6.1%)	13(6.5%)	8(5.7%)	0.763

## Discussion

A lower number of acute appendicitis cases were identified during the pandemic period, this might be due to an increase in the number of self-treated mild conditions. These mild conditions used to get hospitalized, however, in the pandemic era, the fear of contracting the virus might have made patients reluctant to seek care. This finding was previously published by several studies [8–11] including Tankel et al. [11], who emphasized the need for further investigation and research on these patients [11]. Indeed, identifying these patients could spare surgical interventions and unnecessary cost burdens for patients and hospitals.

Another hypothesis could be related to the pathophysiological mechanisms of the diseases. It is believed that acute appendicitis begins as a result of an obstruction at the appendiceal orifice; this obstruction could be due to fecalith, tumor, or lymphoid hyperplasia (infection/inflammation) [12]. This obstruction compromises the blood and lymphatic flow to the appendix leading to ischemia, inflammation, and necrosis [12]. As such, it is reasonable to hypothesize that the decrease in the number of acute appendicitis cases during the COVID-19 pandemic could be due to decreased social contact and eventually decreased microbial exposure.

It is important to note that the cases of AA are circannual with increased cases during the summer months and decreased cases during the winter months [13, 14]. However, collecting the data from the same period from 2019 to 2021 ruled out seasonal variations as a potential cause for this decrease. Finally, further research is needed to rule out the possibility of annual variations. For instance, in the United States, the incidence of acute appendicitis and appendectomies has been decreasing in the last couple of years [15]. Conversely, the incidence of AA is increasing in newly industrialized countries such as Turkey [15].To rule out this cause, similar epidemiological studies should be conducted in Lebanon.

The duration of pre-treatment (or symptoms period) directly correlates with the severity of many surgical and infectious diseases [16]. In our study, the duration of symptoms was not significantly different between the two cohorts. This finding confirms data in published the literature [9, 11, 17]. For instance, both cohorts in the study by Finkelstein et al. had a mean duration of symptoms of 2 days (*p* = 0.5) [9]. Conversely, some studies have shown an increase in the duration of symptoms in the COVID-19 cohort compared to control [10, 18–22]. A study by Delgado-Miguel et al. reported longer symptom progression time (*p* = 0.046) in a pediatric population during the COVID-19 pandemic compared to control. This null comparison in the duration of symptoms in our study could be attributed to several reasons. Namely, a decrease in the magnitude of fear created by the pandemic in our population, also, the fact that the American University of Beirut Medical Center (AUBMC) did not decrease its emergency medical/surgical services during the pandemic period.

It is evident that the management of AA did not change in the study cohort. Most of the patients underwent surgery in the pre-pandemic and pandemic cohorts (87.1% and 91.5% respectively) with no significant difference in either cohort. Additionally, most of the surgical operations were laparoscopic surgeries (76.1% and 78% respectively, *p* = 0.43). This approach to management has been consistent in several studies [9, 11, 17, 22]. In these studies, the number of surgeries in AA patients was not statistically different in the pre-pandemic and pandemic periods, and laparoscopic surgeries were the predominant surgery type. For example, a study of 378 patients, of which 237 had AA pre-pandemic and 141 during the pandemic showed non-statistical differences in conservative and surgical management of AA [11]. Furthermore, Rudnicki et al. showed no change in the percentage of laparoscopic appendectomies, open appendectomies, abdominal drain and antibiotics, and antibiotics only management strategies [22]. The reason why the management strategy of AA did not drastically change in these studies was the preservation of traditional care for these patients by the hospitals to decrease the disease burden of AA.

On the other hand, two studies have shown a significant difference in surgical and conservative treatment of AA between the pre-pandemic and pandemic cohorts [10, 23]. Scheijmans et al. included adult patients with AA from 21 hospitals and showed that conservative management increased between the 2019 and 2020 cohorts from 6.4 to 10.4% (*p* = 0.011). An unsettled debate has been ensued during the pandemic on whether conservative management should be adopted instead of surgeries. Surgery has been shown on several occasions to be more efficacious, a meta-analysis of four RCTs showed significantly higher efficacy for surgery, however complication rates were higher [24]. Non-surgical approaches in AA are not considered viable alternatives to surgery during normal times [24]. However, some would argue that non-surgical approaches would be better f adapted during pandemics to decrease the risk of transmission. Particularly, a meta-analysis of fourteen studies showed that the failure rate of non-operative management (NOM) in AA was 16.4%, and the complication rate after NOM was 4.5% [25]. The authors argue that NOM could be a safe alternative in times of hardship as it has an acceptably low failure and complication rates [25]. Our study serves as an example of intact surgical management in times of pandemic with no delay in pre-treatment presentation. However, further studies are needed to identify the best management strategy for such patients in abnormal situations.

In addition, the imaging modalities adapted in both periods were similar. Most of the patients were diagnosed by CT (86.6% and 88.7% in the pre- pandemic and pandemic cohorts respectively), followed by ultrasound. Diagnostic imaging was also largely unchanged in the study by Hayatghaibi et al. which comprised pediatric patients from the Pediatric Health Information System [8].

The non-delayed emergency department presentation coupled with the predominant surgical management of AA patients during the pandemic led to normal complication rates during this period. Particularly, similar percentages of peri-appendicular abscess, perforation, peri-appendicular fluid, appendicolith, and thickened appendix wall were seen in both cohorts. Similar percentages were seen on CT and Ultrasound. Additionally, no significant increase in abscess, perforation, and gangrene was noticed as part of surgical findings. This particular finding was uncommon, as most of the studies published reported increased complications during the COVID-19 pandemic [9, 10, 17, 22].

Further, surgical, and post-surgical complications did not differ between both cohorts. The number of drains placed in admission complications, re-operations, and post-op abscesses were similar between the cohorts. Additionally, approximately equal numbers of readmissions were seen in the different periods. In short, no extra surgical or post-surgical complications were seen during the pandemic period.

The main limitation of our study is that it is a single-institution, retrospective analysis. Patients’ medical records The records were retrieved using their medical record numbers (MRNs) and data was collated , nonetheless, it is plausible that certain patients or data was overlooked during the process. Additionally, we did not assess the patient’s fear of acquiring COVID-19 nor did we assess the severity of their symptoms. A further limitation to consider is the relatively low sample size and limited timeframe of data collection. Supplemental investigation is required with a larger sample size and broader timeframe. Finally, the literature lacks prospective studies of that sort and thus such studies are needed to get a holistic picture of this subject.

## Conclusion

Our study serves as a set example of how patients presenting for AA were not severely impacted by the COVID-19 pandemic did not significantly affect patients presenting for AA. Despite the COVID-19 related restrictions, Lebanese patients with concerning symptoms were presenting to the emergency department and the American University of Beirut Medical Center was providing them with the standards of care. Studies emphasized on the importance of encouraging patients to seek care when feeling worrying symptoms, as this would decrease health care costs and decrease morbidity [9].However, it is hard to assess whether the benefits of seeking care early in these conditions outweigh the consequences of infection spread. Additional research is needed to decide on the best strategy to adapt in such times. Our study mirrors the Lebanese experience at a tertiary care center and provides an example of a population that focused more on their current symptoms than the fear of acquiring the covid-19 virus.

## Data Availability

The datasets used and/or analyzed during the current study are available from the corresponding author on reasonable request.

## Abbreviations

COVID-19: Coronavirus disease of 2019
ED: Emergency Department
AA: Acute Appendicitis
US: Ultrasound
CT: Computed tomography
MRI: Magnetic resonance imaging
MRNs: Medical record numbers
